# Long-time follow-up of patients with untreated peripheral T cell lymphoma following chidamide combined with cyclophosphamide, epirubicin, vindesine, prednisone, and etoposide therapy: a single-center propensity score-matching study

**DOI:** 10.1007/s12094-023-03135-3

**Published:** 2023-04-05

**Authors:** Chong Wei, Danqing Zhao, Yan Zhang, Wei Wang, Daobin Zhou, Wei Zhang

**Affiliations:** Department of Hematology, Peking Union Medical College Hospital, Chinese Academy of Medical Sciences and Peking Union Medical College, Beijing, 100730 China

**Keywords:** Peripheral T cell lymphoma, Chidamide, Chemotherapy, Treatment response, Survival

## Abstract

**Purpose:**

This is a retrospective, single-center PSM study evaluating the efficacy and safety of chidamide combined with the CHOEP (C-CHOEP) regimen versus the single CHOEP regimen in patients with untreated peripheral T cell lymphomas (PTCL).

**Patients:**

Patients newly diagnosed with PTCL between January 2015 and June 2021 were recruited, and were 1:1 divided into C-CHOEP and CHOEP groups according to their first-line chemotherapy regimens. The PSM method was used to match the baseline variables to balance the confounding factors.

**Results:**

A cohort of 33 patients each in the C-CHOEP and CHOEP groups was generated after propensity score-matching (PSM). The complete remission (CR) rates of the C-CHOEP regimen were higher than that of the CHOEP regimen (56.3 *vs*. 25.8%, *p* = 0.014), whereas the duration of response of the C-CHOEP group was shorter (median DOR 30 *vs.* 57 months), resulting in roughly similar progression-free survival (PFS) and (overall survival) OS between the two groups. The responding patients who received chidamide maintenance therapy showed a trend of superior PFS and OS compared with patients who did not receive maintenance therapy.

**Conclusions:**

The C-CHOEP regimen was well tolerated but failed to show advantages over the CHOEP regimen in patients with untreated PTCL; however, the chidamide maintenance may contribute to a more durable response and stable long-term survival.

## Introduction

Peripheral T cell lymphomas (PTCLs) are a heterogeneous group of mature T cell and natural killer cell neoplasms characterized by poor prognosis and aggressive clinical behavior [1]. PTCLs account for 25–30% of all non-Hodgkin’s lymphomas (NHLs) in China, which is significantly higher than that in Western countries [1–3]. A consensus on the first-line treatment for patients with PTCL has not yet been reached. Anthracycline-based regimens, such as cyclophosphamide, doxorubicin, vincristine, and prednisone (CHOP) or CHOP-like regimens, remain the most commonly used schedules, and their complete remission (CR) rates of approximately 40–50% have been reported [4–6]. However, such regimens have failed to induce sustained remissions, with long-term survival of only 30–40% in most types of PTCLs [1, 3–7].

Evidence regarding more intensive chemotherapies being superior to CHOP is not sufficient. The addition of etoposide to the CHOP (CHOEP) regimen might improve the response rate and event-free survival (EFS) in younger patients (aged ≤ 60 years) [7, 8]. In a previous study, high-dose CHOP alternating with etoposide, cisplatin, cytarabine, and prednisone (ESHAP) followed by autologous stem cell transplantation (auto-SCT) in young patients showed a moderate CR rate (49%) and still failed to achieve better prognoses (4-year progression-free survival [PFS] and overall survival [OS] rates of 30 and 39%, respectively) [9]. In addition, non-anthracycline-based regimens, such as gemcitabine-based chemotherapy, have been explored in several studies for patients with PTCL but still showed non-superior results compared with CHOP-like regimens [10, 11].

Novel drugs, including the histone deacetylase (HDAC) inhibitors, romidepsin, and belinostat, have been approved for patients with relapsed or refractory PTCL in recent years [12, 13]. Chidamide is an innovative class I HDAC inhibitor that was independently designed in China and was approved by the China Food and Drug Administration for treating relapsed or refractory PTCLs. Chidamide monotherapy has been assessed in a multi-center phase 2 trial and real-world studies in China, with an overall response rate (ORR) of 28–39% in relapsed or refractory PTCL [14, 15]. In a phase 1b/2 study evaluating chidamide combined with CHOEP (C-CHOEP) regimen in patients with untreated PTCL (registered with ClinicalTrials. gov, NCT02987244), a modest efficacy was demonstrated with a CR rate of 40.7% and median PFS of 10.7 months [16]. However, the benefit of adding chidamide to the CHOP/CHOEP regimen remains unclear due to the lack of evidences based on phase 3 randomized controlled trials (RCT).

In this study, we aimed to evaluate the benefits of adding chidamide to the CHOEP regimen in patients with untreated PTCLs. The efficacy and toxicity of the two regimens and the long-term survival rates in the two groups were compared. Propensity score-matching (PSM) method was used to balance the baseline characteristics. Moreover, the efficacy of chidamide maintenance therapy and auto-SCT consolidation after the first remission was assessed.

## Methods

### Patients

This was a single-center, retrospective, propensity score-matching study conducted at the Peking Union Medical College Hospital. Patients who were newly diagnosed with PTCL and received the C-CHOEP or CHOEP regimen as the first-line therapy between January 2015 and June 2021 were recruited in this study. The other inclusion criteria were as follows: (1) pathologically confirmed PTCLs, not otherwise specified (PTCL, NOS), anaplastic large cell lymphoma (ALCL), ALK-negative or angioimmunoblastic T cell lymphoma (AITL) according to the World Health Organization (WHO) classification [17]; (2) age between 18 and 70 years; and (3) availability of complete staging and work-up data. All pathological data were reviewed by two independent pathologists to confirm the diagnosis. Patients with subtypes, including ALK-positive ALCL and primary cutaneous ALCL, were excluded from this study.

Patients were divided into C-CHOEP and CHOEP groups according to their first-line chemotherapy regimens. The PSM method was used to match the baseline variables to balance the confounding factors. PSM at a ratio of 1:1 was performed based on the following baseline characteristics: age, Ann Arbor stage, serum lactate dehydrogenase (LDH) level, and pathological subtypes.

The clinical data recorded included age, sex, Ann Arbor stage, B symptoms, Eastern Cooperative Oncology Group (ECOG) performance status, complete blood count, serum LDH level, serum Epstein–Barr virus (EBV) viral load measured by polymerase chain reaction (PCR), bone marrow aspiration and biopsy, computed tomography (CT) and/or positron emission tomography/CT (PET/CT) scan, treatment modalities, treatment responses, survival status, and causes of death. Patients were risk-stratified according to two prognostic scores: the International Prognostic Index (IPI) [18] and the Prognostic Index for PTCL-U patients (PIT) [19].

This study was conducted in accordance with the principles of the Declaration of Helsinki. The study protocols were approved by the institutional review board of Peking Union Medical College Hospital. The requirement for informed consent was waived because anonymized data were used.

### Treatment

The CHOEP regimen included cyclophosphamide (750 mg/m^2^ intravenously on day 1), epirubicin (70 mg/m^2^ intravenously on day 1), vindesine (4 mg intravenously on day 1), prednisone (60 mg/m^2^ orally on days 1–5), and etoposide (100 mg/m^2^ intravenously on days 1–3). The C-CHOEP regimen is composed of chidamide 20 mg twice weekly in addition to the CHOEP regimen and the chidamide was given on day 1 in the first cycle of the CHOEP therapy. Doses and administration schedules other than chidamide in the C-CHOEP regimen were exactly identical to the CHOEP regimen, and chidamide was discontinued during the myelosuppressive period until recovery. The CHOEP and C-CHOEP regimens were repeated every three weeks. Six cycles of the CHOEP or C-CHOEP regimen were scheduled for each patient in both the two groups.

Patients who achieved CR or PR after CHOEP or C-CHOEP induction underwent auto-SCT consolidation. Peripheral blood stem cells were collected after four cycles, and the conditioning regimen consisted of carmustine, etoposide, cytarabine, and melphalan (BEAM) [20]. After CHOEP or C-CHOEP induction with or without auto-SCT consolidation, patients with a PR or CR response underwent chidamide maintenance for 2 years. During maintenance, 20 mg of chidamide twice weekly was administered continuously until disease progression or unacceptable toxicity occurred or the 2 years of course was completed, whichever occurred earlier. The decision to conduct auto-SCT consolidation and chidamide maintenance was made at the physician’s discretion, mainly based on the patient’s age, performance status, economic status, and willingness.

### Assessment of efficacy and adverse events

Efficacy was evaluated after three cycles and one month after the completion of chemotherapy. Medication responses were assessed by chest/abdomen CT and/or PET/CT and were classified as CR, partial remission (PR), stable disease (SD), or progressive disease (PD) according to the 2014 Lugano classification criteria [21]. The ORR was defined as the proportion of patients who achieved CR or PR. Toxicity was graded according to the Common Terminology Criteria for Adverse Events version 4.0.

### Statistical analysis

PFS was defined as the time from diagnosis to the date of disease progression, death from any cause, or the last follow-up, whichever occurred earlier. OS was defined as the time from diagnosis to death from any cause or the last follow-up. Differences between categorical variables were assessed by the χ2 test or Fisher’s exact test. Differences between continuous variables were assessed by the Mann–Whitney U test. OS and PFS analyses were performed by the Kaplan–Meier method. Survival rates between groups were compared by the log-rank test. A *P-*value < 0.05 was considered statistically significant. All analyses were performed using the SPSS software version 20.0 (SPSS Inc., Chicago, IL, USA).

## Results

### Patient characteristics

Ninety eligible patients were identified, including 44 and 46 patients in the C-CHOEP and the CHOEP groups, respectively. Using PSM at a ratio of 1:1, a cohort of 33 patients each in the C-CHOEP and CHOEP groups was generated. Conclusively less than 10% standardized differences in age, Ann Arbor stage, LDH level, and pathological subtypes suggested that these variables were well balanced between the two groups.

All baseline characteristics of the patients in the two groups are listed in Table 1. The median age was 58 (range 17–69) years in the C-CHOEP group and 57 (range 23–70) years in the CHOEP group, respectively. At the time of diagnosis, approximately 90% of the patients had advanced-stage disease in both groups (90.9% in the CHOEP group and 87.9% in the C-CHOEP group, respectively). The proportion of patients in the high-intermediate/high-risk group was 60.6% in the CHOEP group and 54.5% in the C-CHOEP group based on the IPI score (> 2), and was 54.5% and 42.4% based on the PIT score (> 1), respectively. Comparisons of other variables other than age, Ann Arbor stage, LDH level, and pathological subtypes achieved no significant differences further added to the evidences that the two groups were well balanced.

**Table 1:** Clinical characteristics in the two groups after PSM

Characteristics	CHOEP group (*n* = 33)	C-CHOEP group (*n* = 33)	*P-*value
Age, years			
Median (range)	58 (17–69)	57 (23–70)	0.716
> 60	13 (39.4)	12 (36.4)	0.800
Sex, male	21 (63.6)	16 (48.5)	0.215
Ann Arbor stage III/IV	30 (90.9)	29 (87.9)	0.689
B symptoms present Elevated LDH	26 (78.8)	25 (75.8)	0.580
Elevated LDH	24 (72.7)	22 (66.7)	0.415
EBV-DNA ≥ 500 copies/mL	15/32 (46.9)	10/30 (33.3)	0.277
ECOG performance status > 1	11 (33.3)	12 (36.4)	0.796
No. of extranodal involvement > 1	14 (42.4)	11 (33.3)	0.447
Bone marrow involvement	11 (33.3)	7 (21.2)	0.269
IPI score > 2	20 (60.6)	18 (54.5)	0.618
PIT score > 1	18 (54.5)	14 (42.4)	0.325
Histologic subtypes			0.962
PTCL, NOS	11 (33.3)	10 (30.3)	
AITL	16 (48.5)	17 (51.5)	
ALK-negative ALCL	6 (18.2)	6 (18.2)	

*LDH* lactate dehydrogenase, *EBV* Epstein–Barr virus, *ECOG* Eastern Cooperative Oncology Group, *IPI* International Prognostic Index, *PIT* Prognostic Index for PTCL-U patients, PTCL NOS, peripheral T cell lymphoma, not otherwise specified, *AITL* angioimmunoblastic T cell lymphoma, *ALCL* anaplastic large cell lymphoma

### Response

**Fig. 1:**
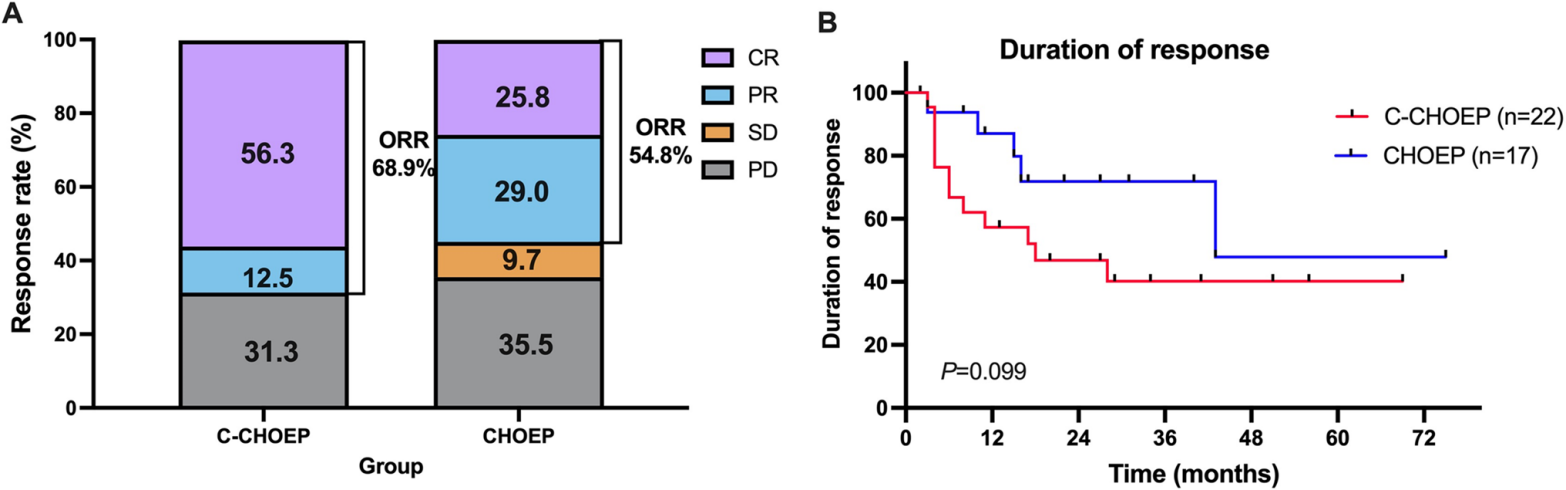
**A** Medication responses in the CHOEP and the C-CHOEP groups; **B** Duration of responses (DOR) in the CHOEP and the C-CHOEP groups

Medication responses in the two groups are summarized in Table 2. In total, 31 patients in the CHOEP group and 32 patients in the C-CHOEP group were evaluable. Significantly higher CR rates were observed in the C-CHOEP group than in the CHOEP group (56.3% [18/32] in the C-CHOEP group *vs.* 25.8% [8/31] in the CHOEP group, *p* = 0.014). The ORRs of the C-CHOEP and CHOEP groups were 68.8% (22/32) and 54.8% (17/31), respectively, attained no significant differences (*p* = 0.256). In addition, a significantly higher rate of CR among responsive patients was achieved with the addition of chidamide to the CHOEP regimen (81.8% [18/22] *vs*. 47.1% [8/17], *p* = 0.037). However, the duration of response (DOR) of the C-CHOEP group was not significantly longer than that of the CHOEP group, with a median DOR of 18 months and 43 months in the C-CHOEP and CHOEP groups, respectively (*p* = 0.099) (Fig. 1).

**Table 2:** Medication responses in the two groups

Response	No. of Patients (%)		
	CHOEP group	C-CHOEP group	*P-*value
No. of patients	31	32	
CR	8/31 (25.8)	18/32 (56.3)	0.014
PR	9/31 (29.0)	4/32 (12.5)	0.190
ORR	17/31 (54.8)	22/32 (68.8)	0.256
CR/ORR	8/17 (47.1)	18/22 (81.8)	0.037
SD	3/31 (9.7)	0/32 (0)	0.113
PD	11/31 (35.5)	10/32 (31.3)	0.722
Auto-SCT consolidation^*^	3/33 (9.1)	10/33 (30.3)	
Chidamide maintenance	3/33 (9.1)	12/33 (36.4)	

*CR* complete remission, *PR* partial remission, *PD* progressive disease, *SD* stable disease, *ORR* overall response rate, *auto-SCT* autologous stem cell transplantation

^*^Patients who underwent auto-SCT after the first remission

After C-CHOEP or CHOEP induction, 15 of the 39 responding patients received chidamide maintenance, including 12 in the C-CHOEP group and 3 in the CHOEP group. 13 patients underwent auto-SCT as consolidation therapy following the first remission, including 10 and 3 responding patients in the C-CHOEP and CHOEP groups, respectively.

### Survival

The median follow-up time was 35 (range 1–81) months in the CHOEP group and 54 (range 2–74) months in the C-CHOEP group. By the end of the follow-up, there were 17 deaths in the CHOEP group (15 disease progression or relapse, 1 pulmonary infection, and 1 gastrointestinal perforation) and 15 deaths in the C-CHOEP group (13 disease progression or relapse, 1 pulmonary infection, and 1 secondary tumor).

**Fig. 2:**
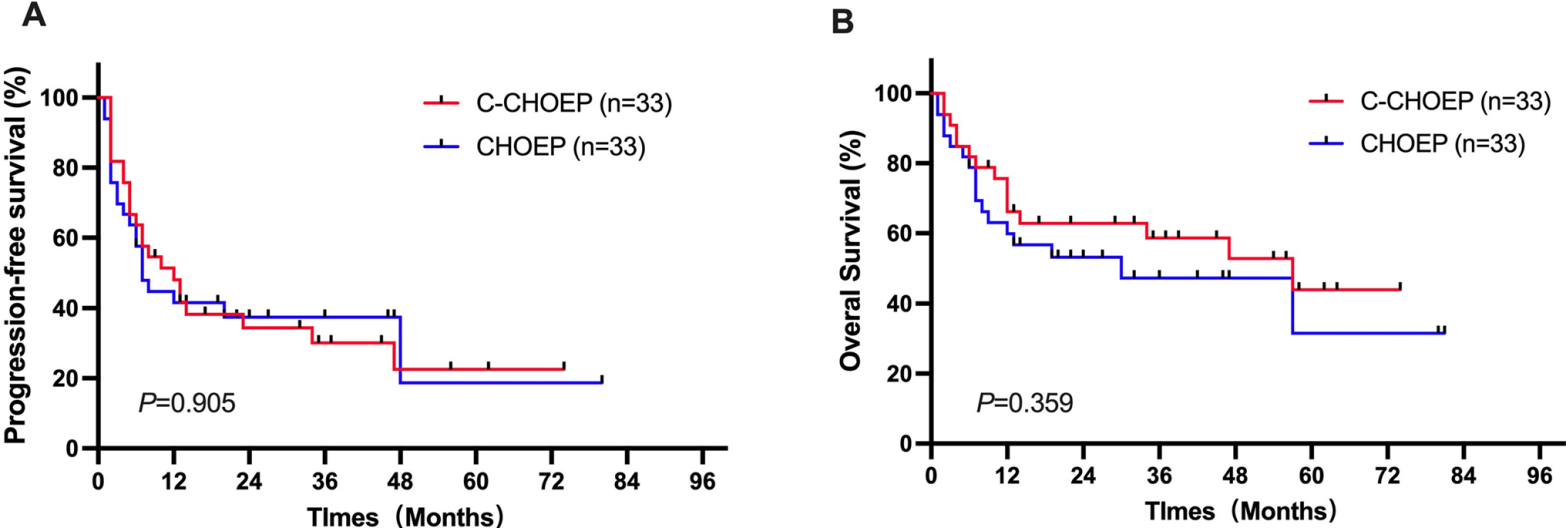
Comparison of progression-free survival (**A**) and overall survival (**B**) between the CHOEP and the C-CHOEP groups

PFS and OS were not significantly different between the two treatment groups (*p* = 0.905 for PFS and *p* = 0.359 for OS). The median PFS in the CHOEP and C-CHOEP groups was 7 months and 12 months (*p* = 0.905), respectively. The 1- and 3-year PFS rates, respectively, were 41.6% and 37.4% in the CHOEP group and 48.1% and 30.1% in the C-CHOEP group (Fig. 2A). The median OS in the CHOEP and C-CHOEP groups was 30 months and 57 months (*p* = 0.359), respectively. The 1- and 3-year OS rates, respectively, were 59.9% and 47.2% in the CHOEP group and 66.2% and 58.7% in the C-CHOEP group (Fig. 2B).

**Fig. 3:**
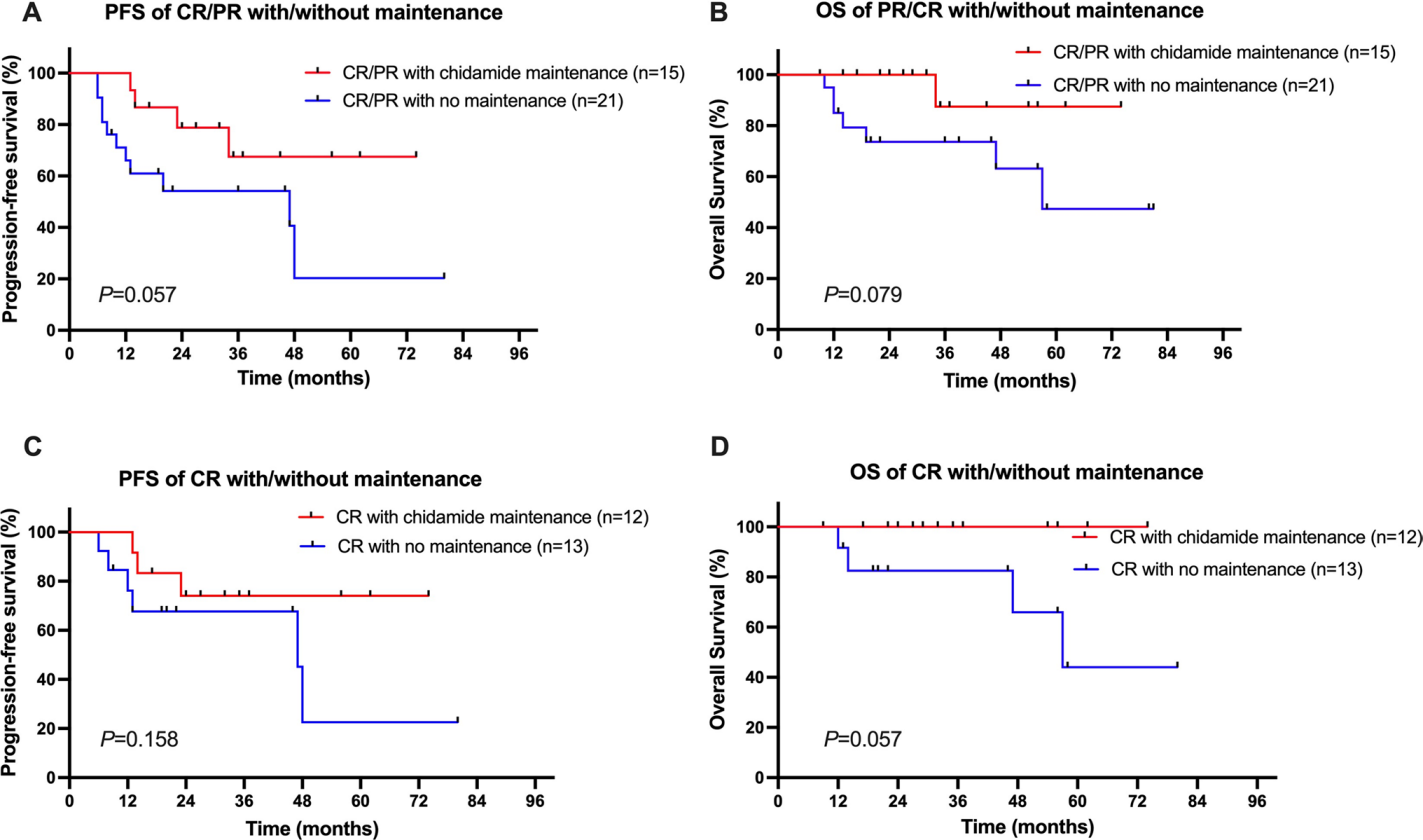
Survival of the patients with or without chidamide maintenance therapy. Comparisons of the PFS (**A**) and the OS (**B**) between CR/PR patients after first-line chemotherapy with or without chidamide maintenance therapy. Comparisons of the PFS (**C**) and the OS (**D**) between CR patients after first-line chemotherapy with or without chidamide maintenance therapy

**Fig. 4:**
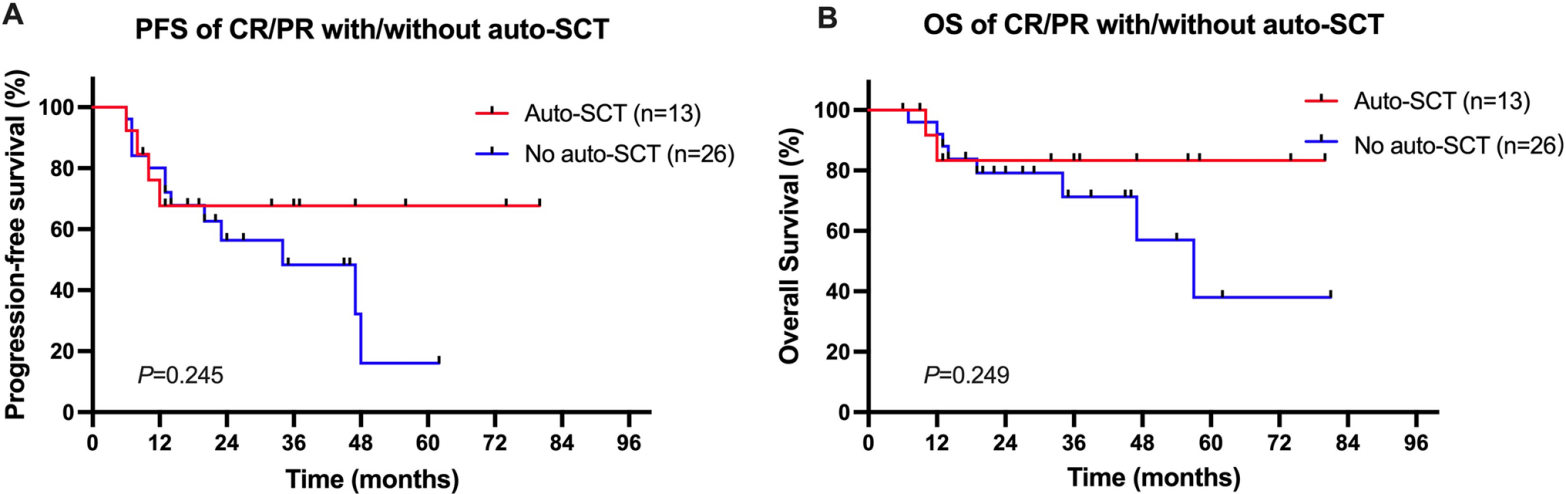
Survival of the patients with or without auto-SCT consolidation. Comparisons of the PFS (**A**) and the OS (**B**) between CR/PR patients after first-line chemotherapy with or without auto-SCT consolidation

The benefits of chidamide maintenance therapy and auto-SCT consolidation after the first remission were further analyzed. Although there were no statistically significant differences, responding patients (CR/PR) who received chidamide maintenance therapy showed a trend of superior PFS and OS compared with patients who did not receive maintenance therapy, with 3-year PFS rates of 67.5% in the chidamide maintenance group versus 54.2% in the no maintenance group, and 3-year OS rates of 87.5% versus 73.3%, respectively (Fig. 3). In addition, the value of auto-SCT consolidation was also evaluated in the responding patients (CR/PR). The median PFS and OS were not reached in patients who underwent auto-SCT after the first remission, whereas they were 34 and 57 months in those who did not undergo auto-SCT consolidation. A plaque survival curve was reached at 1 year for the auto-SCT group, compared with a continuous decline in patients who did not receive auto-SCT consolidation (Fig. 4); however, such differences were still not statistically significant (*p* = 0.245 for PFS and *p* = 0.249 for OS).

### Adverse events

Table 3 listed all grade 3–5 adverse events (AEs) in the two groups. Overall, the C-CHOEP regimen was well tolerated and no significantly severe hematologic and non-hematologic toxicities were observed compared to the CHOEP regimen. The most common AEs in the both two groups were hematologic toxicities. The incidence of grade 3–4 neutropenia was 57.6% in the CHOEP group and 60.6% in the C-CHOEP group (*p* = 0.802). Non-hematologic toxicities were mainly grade 1–2. 2 serious pulmonary infections were observed: one patient suffered pneumocystis pneumonia in the C-CHOEP group, and the other patient suffered a mixed bacterial and fungal infection in the CHOEP group. 1 patient (3.0%) in the C-CHOEP group was allergic to etoposide wih a grade 3 and the administration of which was adjusted in the subsequent courses. Of note, EBV or hepatitis B virus (HBV) reactivation was not observed in the both two groups.

**Table 3:** AEs in the two groups

Toxicity	CHOEP (*n* = 33)			C-CHOEP (*n* = 33)			*P-*value
	Grade 3	Grade 4	Grade 5	Grade 3	Grade 4	Grade 5	
Neutropenia	9 (27.2)	10 (30.3)	0 (0)	7 (21.2)	13 (39.4)	0 (0)	0.802
Anemia	4 (12.1)	0 (0)	0 (0)	5 (15.2)	0 (0)	0 (0)	0.720
Thrombocytopenia	7 (21.2)	0 (0)	0 (0)	8 (24.2)	0 (0)	0 (0)	0.769
ALT elevation	1 (3.0)	0 (0)	0 (0)	1 (3.0)	0 (0)	0 (0)	1.000
Infection	1 (3.0)	0 (0)	1 (3.0)	1 (3.0)	1 (3.0)	1 (3.0)	0.642
Allergy	0 (0)	0 (0)	0 (0)	1 (3.0)^*^	0 (0)	0 (0)	1.000
Nausea/Vomiting	1 (3.0)	0 (0)	0 (0)	1 (3.0)	0 (0)	0 (0)	1.000

*ALT* alanine aminotransferase

^*^Allergic to etoposide

During the chidamide maintenance period, most patients were well tolerated. The most common AE was hematologic toxicity. Grade 3–4 AEs included neutropenia (13.3%, 2/15), thrombocytopenia (13.3%, 2/15), and anemia (6.7%, 1/15). 2 patients reduced their chidamide to 15 mg as a result of neutropenia and thrombocytopenia, respectively.

## Discussion

In contrast to the progression in the treatment of aggressive B cell lymphoma, the medication management of patients with PTCL has been disappointing, with no major progression has been made over the past decades. Thus far, no consensus on the standard therapy for PTCL has been reached due to the lack of evidences based on the RCTs and the geographic variation in the incidences of the disease. CHOP or CHOP-like regimens remain the most widely used first-line therapy. Despite more intensive chemotherapy and gemcitabine-based regimens have been investigated, advantages over the conventional CHOP regimen still failed to show [9–11, 22]. Numerous previous studies have suggested that the use of cytotoxic chemotherapies at varying intensities and densities is insufficient for the treatment of PTCL. Thus, novel targeted therapies are urgently required. In this context, The ECHELON-2 trial compared the efficacy between combining brentuximab vedotin (BV) with cyclophosphamide, doxorubicin, and prednisone (CHP) and the CHOP regimen in previously untreated CD30 + PTCLs; PFS and OS were superior in the BV + CHP arm, leading to FDA approval for this regimen in frontline settings [23].

Epigenetic dysregulation plays an important role in the PTCL pathogenesis. In the recent years, several HDAC inhibitors, such as belinostat, romidepsin, and chidamide, have been approved to treat relapsed or refractory (R/R) PTCLs. On the whole, the effective rate of a single agent of these HDAC inhibitors is approximately 25–40% in patients with R/R PTCLs [12–15]. Furthermore, the safety and the efficacy of HDAC inhibitors combined with the CHOP regimen have also been assessed in several clinical trials. In a randomized phase III study of romidepsin plus CHOP versus only CHOP in patients with untreated PTCLs, the introduction of romidepsin did not improve the response rate, PFS, or OS, whereas increased the frequency of grade ≥ 3 AEs [24]. In another phase 1b/2 study evaluating chidamide combined with the CHOEP regimen in patients with untreated PTCLs, such regimen was generally well tolerated with modest efficacy (CR rate of 40.7% and ORR of 60.2%), revealing no clear benefit of adding chidamide to CHOEP due to the lack of a control arm [16].

Based on these findings, we performed a single-center, retrospective PSM study to further evaluate the benefits of adding chidamide to the CHOEP regimen in patients with untreated PTCL. In brief, the C-CHOEP regimen did not have significant advantages over the CHOEP regimen in patients with untreated PTCL. Although the CR rate and ORR were improved with the addition of chidamide to the CHOEP regimen, the higher CR rates and ORRs did not contribute to prolonged response duration, resulting in roughly similar PFS and OS between patients with or without chidamide. Regarding the safety profile, the C-CHOEP regimen was generally well tolerated. No significant increase in grade ≥ 3 adverse events or the incidence of EBV/HBV reactivation was observed. Since the IPI was found to have prognostic significance in the survival of the patients with newly diagnosed PTCL, and the choice of chemotherapy regimen may make a difference to the survival outcome, a possible explanation for the non-significantly different survival outcome may be attributed to the less benefit of C-CHOEP in patients with low IPI, which was consistent with other studies conducted in Chinese patients [25, 26].

Genes that regulate DNA methylation, such as TET2, DNMT3A, and IDH1/2, are found to be prevalent in PTCL, especially AITL. Demethylating agents may also play a therapeutic role in PTCL treatment. Recent data have demonstrated that the combination of oral 5-azacytidine and romidepsin is highly effective in treating patients with PTCL [27]. Prompted by the encouraging results that showing the efficiency of double epigenetic regulating agents, a multi-center phase III trial evaluating the combination of chidamide, 5-azacytidine, and CHOP regimen versus only CHOP in patients with untreated PTCL is under recruitment in our center (NCT05075460). In this context, since the present study found that the addition of chidamide has edged favorable effect and with no more severe AEs, the combination of chidamide, 5-azacytidine, and CHOP regimen is highly expected.

On the other hand, although a fair number of PTCL patients are sensitive to chemotherapy, their response duration is rather short, with frequent relapses that often result in poor long-term outcomes. The relapse rate was reported to be approximately 30% in PTCL, which is generally higher than that in B cell lymphoma [5, 28]. In our study, the responding patients who received chidamide maintenance therapy showed a trend of superior PFS and OS compared with patients who did not receive maintenance therapy. This finding indicates that the chidamide maintenance therapy after the first remission is feasible and could induce a more durable response and stable long-term survival. Chidamide is an oral tablet with the advantage of being convenient to use, and the AEs of chidamide maintenance are mainly mild hematologic toxicities. Previously, long-term treatment with romidepsin and pralatrexate was only reported in case reports [29, 30]. To the best of our knowledge, this is the first study to evaluate the importance of HDAC inhibitors used as maintenance therapy in patients with untreated PTCL.

Auto-SCT might play a role as frontline therapy in PTCL by increasing CR rates and reducing relapses. In a study of the Nordic Lymphoma Group (NLG T-01 study), the largest prospective trial evaluating upfront auto-SCT in PTCL, the outcome was encouraging, with a 5-year PFS rate of 44% [20]. Cumulative evidences from other studies, including a large population-based retrospective study from the Swedish Lymphoma Registry and a prospective cohort study from the COMPLETE registry, further support upfront auto-SCT for eligible patients with PTCL [7, 31]. In the present study, a trend toward improved long-term survival was observed in the auto-SCT consolidation group with a longer median OS and a plaque survival curve at 1 year. A better definition of the benefit of upfront auto-SCT in patients with PTCLs should be further evaluated in prospective randomized trials.

The limitations of our study must be acknowledged. Due to the rarity of PTCL, conducting RCT is relatively difficult. Although we utilized the PSM method to mimic RCT, the results may still be confounded by selection bias and unbalanced clinical characteristics; however, such confounding factors were expected to bias the results toward null, rather than caused spurious associations. The results of our study may serve as a reference for future multi-center, prospective, randomized trials; in addtion, differences in the responses and survival among different subtypes of PTCL could also be investigated in future large-scale studies.

## Conclusion

In conclusion, this study demonstrated that the C-CHOEP regimen was generally well tolerated but failed to show advantages over the CHOEP regimen in patients with untreated PTCL during long-term follow-up. However, chidamide maintenance therapy may contribute to a more durable response and stable long-term survival in patients who achieve CR/PR after induction therapy, and the responding patients may also benefit from auto-SCT consolidation. Conservatively, this study adds to the evidence that chidamide could be a promising supplement to combined medication regimen for treating PTCL and provide baseline data for further prospective randomized trials to explore other options, for instance the combination of immunosuppressive agent.

## Data Availability

The data generated in this study are available upon request from the corresponding author.
